# An update on the prevalence of low back pain in Africa: a systematic review and meta-analyses

**DOI:** 10.1186/s12891-018-2075-x

**Published:** 2018-06-21

**Authors:** Linzette Deidrè Morris, Kurt John Daniels, Bhaswati Ganguli, Quinette Abegail Louw

**Affiliations:** 1 Division of Physiotherapy, Department of Health and Rehabilitation Sciences, Faculty of Medicine and Health Sciences, Stellenbosch University, PO BOX 241, Cape Town, 8000 South Africa; 2 Division of Epidemiology and Biostatistics, Faculty of Medicine and Health Sciences, Stellenbosch University, Tygerberg, South Africa; 3 Department of Statistics, University of Calcutta, Kolkata, India

**Keywords:** Low back pain, Africa, Prevalence, Epidemiology, Systematic review, Meta-analysis

## Abstract

**Background:**

Low back pain (LBP) remains a common health problem and one of the most prevalent musculoskeletal conditions found among developed and developing nations. The following paper reports on an updated search of the current literature into the prevalence of LBP among African nations and highlights the specific challenges faced in retrieving epidemiological information in Africa.

**Methods:**

A comprehensive search of all accessible bibliographic databases was conducted. Population-based studies into the prevalence of LBP among children/adolescents and adults living in Africa were included. Methodological quality of included studies was appraised using an adapted tool. Meta-analyses, subgroup analyses, sensitivity analyses and publication bias were also conducted.

**Results:**

Sixty-five studies were included in this review. The majority of the studies were conducted in Nigeria (*n* = 31;47%) and South Africa (*n* = 16;25%). Forty-three included studies (66.2%) were found to be of higher methodological quality. The pooled lifetime, annual and point prevalence of LBP in Africa was 47% (95% CI 37;58); 57% (95% CI 51;63) and 39% (95% CI 30;47), respectively.

**Conclusion:**

This review found that the lifetime, annual and point prevalence of LBP among African nations was considerably higher than or comparable to global LBP prevalence estimates reported. Due to the poor methodological quality found among many of the included studies, the over-representation of affluent countries and the difficulty in sourcing and retrieving potential African studies, it is recommended that future African LBP researchers conduct methodologically robust studies and report their findings in accessible resources.

**Trial registration:**

The original protocol of this systematic review was initially registered on PROSPERO with registration number CRD42014010417 on 09 July 2014.

## Background

Low back pain (LBP) is arguably the most prevalent musculoskeletal condition found among both developed and developing nations [1–4]. Broadly defined as pain or discomfort in the lumbar region of the spine [1, 2]; LBP is the leading cause of activity limitation, results in significant losses in productivity at work and incurs billions of dollars in medical expenditure annually [1, 3, 4]. The prevalence of LBP worldwide is estimated to be between 30 and 80% among the general population and has been found to increase with age [5]. In addition, a higher prevalence of LBP has been associated with a lower socioeconomic status and lower education levels [5, 6]. According to the Global Burden of Disease (GBD) 2010 study, LBP is currently the sixth highest burden on a list of 291 conditions and is the cause of more years lived with disability (YLDs) globally than any other disease [4]. Affecting just about anyone, of any gender, race or socioeconomic background [6], LBP has a substantial impact on the overall and financial well-being of an individual and society [5, 7]. Therefore, it was postulated that the burden of LBP would be greater in lower and middle income countries (LMICs) like those situated in Africa [7, 9]. A systematic review published in 2007 revealed that the prevalence of LBP in Africa was comparable to that of developing nations, and was rising [10].

Despite the GBD 2010 and World Health Organization (WHO) reports [4, 8, 9], and coupled with the high prevalence of LBP in Africa [10]; LBP and other musculoskeletal conditions remain less prioritized in LMICs, due to more pressing health issues like HIV/AIDS [3]. This is most likely due to the fact that although LBP causes significant disability and related health costs, it is not life-threatening [4, 11]. LBP however remains a global health concern and an immense burden for LMICs, such as those in Africa where health budgets are already restricted and channelled to other higher priority conditions [1, 2, 5, 7]. Of concern is that due to various epidemiologic challenges faced in various LMICs in Africa and the subsequent lack of accurate data, the true burden of LBP is still not well understood or known. In the 7 years since the previous review was published, a large number of studies have emerged. The following paper therefore reports on an updated search of the current literature into the prevalence of LBP among African nations (children, adolescents, adults; males and females). It was hoped that a better understanding of the current burden of LBP in African LMICs would be established. Furthermore, this paper also highlights the specific challenges faced in retrieving epidemiological information in Africa and on conducting meta-analyses of LBP data, as well as the methodological shortcomings of published African studies.

## Methods

The MOOSE (Meta-analysis Of Observational Studies in Epidemiology) were used [12]. The protocol for this updated review was registered on PROSPERO prior to commencement (protocol registration number: CRD42014010417) [13].

Studies had to primarily report on the prevalence of LBP among nations situated on the African continent were included. Studies could report on the prevalence of musculoskeletal conditions as a whole, yet had to provide subgroup data for LBP prevalence. Studies could report on the following recall periods for LBP prevalence, namely: point, annual or lifetime prevalence. Subjects included in the studies could be any race, gender and age. Studies could be published in English, Afrikaans or French, since these are three of the most common languages in which scholarly communication in Africa is conducted [14]. French studies were translated by a French-speaking African native. To validate the translations, we cross-checked the French translations with the English abstract of the article (which is typically available online) to check for any marked discrepancies and reverse translations were done to ensure validity of translations. Dissertations, conference proceedings, commentaries/letters and other grey literature were excluded from this review.

A comprehensive update of the previous search [10] was conducted in the following bibliographic databases via the Stellenbosch University’s library website: *EbscoHost (including CiNAHL, Africa-Wide Information, Health Source: Nursing/Academic edition, SPORTDiscus), Medline, ScienceDirect, Scopus, PEDro, PubMed, SA ePublications, Cochrane Library, ProQuest Medical Library, African Journals Online (AJOL)* and *Web of Science.* The main search terms were: *low back pain, Africa* and *prevalence.* The original search strategy was revised where necessary and excluded *management* and *rehabilitation.* The full search strategy is available on request from the corresponding author. Secondary searching (PEARLing) was conducted (PEARLing is a search method whereby the reference lists of all included and excluded studies are searched for other studies which may not have been identified during the database search). Manual searching was not conducted due to the difficulty in replicating this method. The search was commenced and conducted between June 2014 and October 2014, and an updated search was conducted in March 2015 and July 2016. A final search was conducted in April 2017, prior to submission. Articles published and indexed from inception of the databases to the end of the search period were included.

The titles and abstracts of all potentially relevant population-based studies were screened by two reviewers independently. Methodological appraisal of included studies was conducted using the same critical appraisal tool as in the original review [10, 15]. The tool was however further adapted for use in this review (Table 1), by reducing the previous items 7, 8 and 9 to one item (7a- c), as all these items pertained to the validation of the data collection tool used in the study. For the purposes of this review, all items in the appraisal tool were equally weighed and the total score for the tool was 10. No subminimum criteria were applied.

**Table 1:** Methodological appraisal tool for LBP prevalence studies (adapted) [15]

Criteria	Yes/No	Comments
Is the final sample representative of the target population?		
1. At least 1 of the following must apply in the study: an entire target population, randomly selected sample or sample stated to represent the target population.		
2. At least 1 of the following: reasons for non-response described, non-responders described, comparison of responders and non-responders, or comparison of sample and target population.		
3. Response rate, and if applicable, drop-out rate reported		
Quality of data		
4. Were the data primary data of LBP, or was it taken from a survey not specifically designed for that purpose?		
5. Were the data collected from each subject directly or were they collected from a proxy?		
6. Was the same mode of data collection used for all subjects?		
7. At least 1 of the following in case of: a) Questionnaire: a validated questionnaire or at least tested for reproducibility? b) Interview: interview validated, tested for reproducibility, or adequately described and standardized? c) Examination: examination validated, tested for reproducibility, adequately described and standardized?		
Definition of LBP		
8. Was there a precise anatomic delineation of the lumbar area or reference to an easily obtainable article that contains such specification?		
9. Was there further useful specification of the definition of LBP, or question(s) put to study subjects quoted such as frequency, duration, or intensity, and character of the pain. Or was there reference to an easily obtainable article that contains such specification?		
10. Were the recall periods clearly stated: e.g. 1 week, 1 month, lifetime?		
Total score (10)		

Appraisal of studies was conducted independently by two reviewers. Studies scoring 60% or less on the appraisal tool were deemed as low quality studies and were excluded from the meta-analyses. The 60% cut-off was deemed appropriate based on the fact that no subminimum criteria were applied due to the heterogeneous nature of LBP data and that the average methodological score of all studies was 66%. It was therefore decided that all studies which were below the average score were relatively lower in methodological quality compared to the rest of the included studies.

Data were extracted using specifically-designed extraction sheets and were entered into Microsoft (MS) Excel spreadsheets [16]. The following data were extracted from included studies: *author name(s), year of publication, country of publication, study design, data collection tool/outcome measure tool(s), population, study setting (including if rural or urban setting), sample size, age group/age (range and/or mean ± standard deviation), gender, data collection period, LBP definition, LBP recall period, reliability/validity of measurement tools, response rates* and *LBP prevalence rates (point, annual and lifetime).*

From the data extracted, the pooled point, annual and lifetime prevalence (summary estimates) of LBP among African nations, as well as the 95% confidence intervals (CI), were calculated for conducting meta-analyses of observational data. A random effects model to adjust for heterogeneity was used since LBP data inherently varies between studies due to differences in risk factors and characteristics between populations. Sub-group analyses were conducted for age group (adults and children/adolescents), country status (low income, low middle income and upper middle income), gender (male and female) and setting (community, industry, hospital, professional and school). Sensitivity analyses were conducted to assess if the inclusion of the lower methodological quality studies would change the results of the analyses. Publication bias was also assessed using Duval and Tweedie’s Trim and Fill method [81].

## Results

**Fig. 1:**
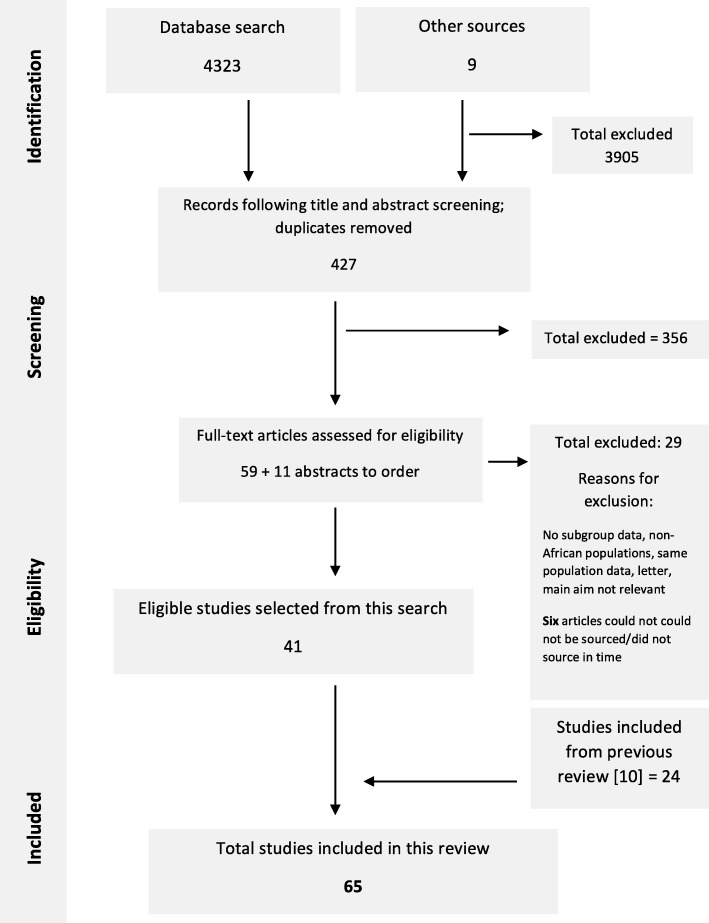
Flow chart depicting study selection procedure

The results of the comprehensive updated search of literature into the prevalence of LBP in Africa are depicted in Fig. 1. A total of 65 studies were included in this review (of which 40 were published after the original review was conducted) [17–70, 81–91]. A list of the excluded studies and the reasons for their exclusion is available from the corresponding author.

### General description of included studies

More than 72.3% of the included studies were conducted in lower income and lower middle income countries [17, 21, 22, 25–29, 31–34, 36, 37, 40–44, 46–48, 50–53, 57–60, 63–67, 69, 83–91]. The majority of the studies were conducted in Nigeria, which is a lower middle income country (*n* = 31; 47.7%) [22, 25, 27, 29, 32, 33, 36, 41, 44, 46–48, 50, 52, 53, 57–60, 64–66, 81–83, 86–91] and South Africa, which is an upper middle income country (*n* = 16; 24.6%) [19, 20, 23, 24, 30, 35, 38, 39, 45, 49, 54–56, 61, 62, 70]. Three of the included studies were published in the French language [18, 26, 51], the rest were published in English. Fifteen (27.8%) of the 54 independent African countries (countries as recognised by the United Nations) are represented in this review. Forty-five studies included both male and female participants (75%) [17, 18, 20–22, 27–37, 40, 46, 48–53, 55, 57–59, 61, 62, 67–70, 81, 82, 84–86, 89, 90]. Fourteen of the included studies included children and/or adolescents between the ages 11 and 19 years (21.5%) [17–19, 31, 34, 35, 41, 44, 53, 56, 59, 67, 69, 85]. The response rates were reported by 72.3% of the studies (*n* = 47) [19–25, 27, 28, 30–41, 46–50, 52–54, 59, 61–64, 66–70, 82, 85–91] and ranged from 11 to 100%. Forty-two of the studies were conducted in an urban setting (64.6%), while nine studies (13.8%) where conducted in a rural setting. The rest of the studies (*n* = 14; 21.5%) were conducted in a setting which incorporated both rural and urban communities.

The most common study design was cross-sectional (*n* = 60; 92.3%). Two studies used a prospective study design [17, 42] and three used a retrospective study design [21, 26, 84]. Most of the included studies used questionnaires. Three studies reviewed medical records [21, 26, 84], and eight studies included a physical examination [17, 40, 42–45, 47, 58]. Twelve studies conducted interviews [17, 20, 40–44, 54, 55, 57, 58, 60]. It was unclear in three of the studies which sampling method was used [19, 50, 59]. Nine studies did not explicitly provide a clear recall period (point, lifetime or annual) for LBP (15%) [17, 41, 42, 44, 45, 49, 70, 71, 84]. Two studies used the index pregnancy (up to 40 weeks) as the recall period [60, 83].

The most common population studied was health professionals and hospital staff (*n* = 17; 26.2%) [22, 28, 30, 37, 45, 46, 48–52, 55, 61–63, 82, 91]. Health professions studies included physiotherapists, general surgeons, dentists, nurses, general surgeons and oral hygienists. Workers were studied in 21 of the eligible studies (32.3%) [20, 24, 26, 27, 32, 33, 36, 38, 39, 43, 44, 47, 54, 57, 64–66, 81, 87–90] and included the following sectors: commercial, industry, transport and farming. Computer-users were only studied in one included study [36] and two studies included sports players [19, 56]. The sports players studied were cricketers. One study reported on LBP prevalence among school teachers [68]. An overview summary of the descriptive data extracted from the included studies is provided in Table 2.

**Table 2:** General description of included studies (*n* = 65)

Study ID	Year	Country	Population description	Study setting	Design/tool	Sampling method	Age (years)	Gender	n	RR
Mulimba [17]	1990	Nairobi	Private patients	Private clinic	P; I/E	population	11–75	F/M	2201	NP
Bezzaoucha [18]	1992	Algiers	General population	Community	C; Q	population	15 and over	F/M	6956	NP
Harris [19]	1993	South Africa	Cricketers	Cricket clubs/schools	C; Q	unclear	15–35	M	110	90
Schierhout et al. [20]	1993	South Africa	Factory workers	Factories	C; I	block random	18 and older	F/M	155	100
Mijiyawa et al. [21]	2000	Togo	OPD patients	Rheumatology clinic	R; MR	population	17–94	F/M	9065	100
Omokhodion et al. [22]	2000	Nigeria	Hospital staff	Hospital	C; Q	population	20–60	F/M	74	93
Worku [23]	2000	Lesotho	Mothers	Community	C: Q	stratification	18 and older	F	4001	100
Wallner-Schlotfeldt et al. [24]	2000	South Africa	Material handlers	Industry	C; Q	population	23–59	M	126	68
Omokhodion et al. [25]	2002	Nigeria	General population	Community	C; Q	stratification	20–85	F/M	900	100
Mbaye et al. [26]	2002	Senegal	Public transport employees	Industry	R; MR	population	18–55	M	1500	NP
Omokhodion et al. [27]	2003	Nigeria	Civil service workers	Corporate	C; Q	stratification	20–60	F/M	840	66
Igumbor et al. [28]	2003	Zimbabwe	Physiotherapists	Physiotherapy practices	C; Q	population	23–76	F/M	107	72
Omokhodion et al. [29]	2004	Nigeria	General population	Community	C; Q	stratification	20–82	F/M	474	NP
Govender [30]	2004	South Africa	Nurses	Hospital	C; Q	random	20–62	F/M	320	68
Prista et al. [31]	2004	Mozambique	School children	Schools	C; Q	stratification	11–16	F/M	204	85
Fabunmi et al. [32]	2005	Nigeria	Peasant farmers	Farm settlement	C; Q	multi-stage	18 and older	F/M	500	100
Sanya et al. [33]	2005	Nigeria	Industrial workers	Industry	C; Q	population	20–60	F/M	604	53
Bejia et al. [34]	2005	Tunisia	School children	Schools	C; Q	random	11–19	F/M	622	98
Jordaan et al. [35]	2005	South Africa	Adolescents	Schools	C; Q	stratified cluster	13–18	F/M	1004	89
Adedoyin et al. [36]	2005	Nigeria	Computer users	University campus	C; Q	convenience	29 ± 2.5	F/M	1041	93
Bejia et al. [37]	2005	Tunisia	Hospital staff	Hospital	C; Q	random	18–60	F/M	350	100
Van Vuuren et al. [38]	2005	South Africa	Steel plant workers	Industry	C; Q	population	31.76 ± 7.80	M	366	96
Van Vuuren et al. [39]	2005	South Africa	Manganese plant workers	Industry	C; Q	convenience	35.2 ± 9.29	M	109	100
Galukande et al. [40]	2005	Uganda	OPD patients	OPD Clinic	C; Q/E	population	19–86	F/M	1033	100
Ayanniyi et al. [41]	2006	Nigeria	Pregnant females	Antenatal clinics	C; I	consecutive	12–45 (26.95 ± 5.37)	F	2187	88
Hill et al. [42]	2007	Ghana	Community women	Hospital	P; I/E	convenience	18 and older	F	1328	NP
Bio et al. [43]	2007	Ghana	Gold miners	Gold mines	C; I/E	simple random	27–53/(40 ± 5.6)	M	280	NP
Balogun and Owoaje [44]	2007	Nigeria	Female traders	Trade market	C; I/E	population	16–80/(37.3 ± 12.8)	F	281	NP
Naidoo and Coopoo [45]	2007	South Africa	Nurses	Public hospitals	C; Q/E	volunteered	37	F	107	NP
Odebiyi et al. [87]	2007	Nigeria	Commercial/private drivers	Industry	C; Q	unclear	30 and older	M	500	100
Adegoke et al. [46]	2008	Nigeria	Physiotherapists	2°and 3° hospitals	C; Q	population	22–57 (33.7 ± 6.8)	F/M	126	58
Akinbo et al. [47]	2008	Nigeria	Commercial drivers/cyclists	Commercial driver garages	C; Q/E	random	37.1 ± 10.5 / 31.13 ± 8.13	M	599	75
Sikiru and Shmaila [48]	2009	Nigeria/Ethiopia	Nurses	Specialized hospitals	C; Q	population	33.69 ± 8.83	F/M	508	82/83
Booysens et al. [49]	2009	South Africa	Oral hygienists	Dental practices	C; Q	population	20 and older	F/M	362	38
Isa et al. [81]	2009	Nigeria	Commercial motorcyclists	Industry	C; Q	convenience	21–50	F/M	600	NP
Tinubu et al. [50]	2010	Nigeria	Nurses	Private / public hospitals	C; Q	unclear	22–58(36.4 ± 7.75)	F/M	118	80
Ouédraogo et al. [51]	2010	Burkina Faso	Hospital workers	Tertiary hospital	C; Q	consecutive	22–58 (38 ± 8.25)	F/M	436	NP
Sikiru and Hanifa [52]	2010	Nigeria	Nurses	Specialized hospitals	C; Q	volunteered	25–55 (39.20 ± 9.09)	F/M	408	82
Abiodun- Solanke et al. [82]	2010	Nigeria	Dentists/ dental auxiliaries	Dental hospitals	C;Q	cluster random	21–60	F/M	210	77.3
Ayanniyi et al. [53]	2011	Nigeria	Adolescents (school children)	Schools	C; Q	cluster random	10–19 (15.0 ± 1.7)	F/M	3185	72
Saidu et al. [64]	2011	Nigeria	Factory workers	Factories	C; Q	convenience	21–58	F/M	420	84
Himalowa and Frantz [54]	2012	South Africa	Manual construction workers	Construction sites	C; I	population	17–65 (31.9 ± 10.7)	M	212	100
Desai et al. [55]	2012	South Africa	General surgeons	University	C; I	population	33.57 ± 6.48	F/M	76	NP
Noorbhai et al. [56]	2012	South Africa	Adolescent cricket players	Top cricketing schools	C; Q	purposive	14–17 (15.1 ± 1)	M	234	NP
Birabi et al. [57]	2012	Nigeria	Peasant farmers	Farm settlement	C; I	cluster random	18–58(36.71 ± 8.98)	F/M	310	NP
Ogunbode et al. [58]	2013	Nigeria	Adult patients	Family practice clinic	C; I/E	population	18–85 (42.5 ± 15.5)	F/M	485	NP
Oyeyemi et al. [83]	2013	Nigeria	Pregnant females	Teaching Hospital	C;Q	convenience	25.61 ± 5.02	F	310	NP
Akinpelu et al. [59]	2013	Nigeria	Adolescent students	Community	C; Q	unclear	12–17	F/M	900	90
Jimoh et al. [60]	2013	Nigeria	Pregnant females	Antenatal care clinics	C; I	population	29.93 ± 4.80	F	200	NP
Madiba et al. [61]	2013	South Africa	Nurses	Tertiary hospital	C; Q	purposive	29–65	F/M	125	74
Tella et al. [65]	2013	Nigeria	Peasant farmers	Farms	C; Q	convenience	unclear	F/M	604	NP
Rufa’i et al. [66]	2013	Nigeria	Professional drivers	Motor parks	C;Q	convenience	19–64	M	200	86.3
Botha et al. [62]	2014	South Africa	Dentists	Dental practices	C; Q	random	45 ± 13	F/M	338	11
El-Soud et al. [63]	2014	Egypt	Nurses	Tertiary hospitals	C, Q	Population	18 and older	F/M	150	100
Chiwaridzo et al. [67]	2014	Zimbabwe	Adolescents	Government schools	C; Q	cluster random	13–19	F/M	544	97.8
Erick and Smith [68]	2014	Botswana	School teachers	Schools	C; Q	cluster random	38.5 ± 8.62	F/M	1747	56.3
Mwaka et al. [69]	2014	Uganda	Pupils	Schools	C; Q	cluster random	Mean 13.6	F/M	532	67.9
Major-Helstoot et al. [70]	2014	South Africa	General population	Communities	C; Q	cluster random	44.8 ± 13.95	F/M	489	97
Akodu et al. [88]	2014	Nigeria	Traffic wardens	Traffic centres	C; Q	unclear	38.22 ± 2.98	F/M	187	82
Triki et al. [84]	2015	Tunisia	Children/adolescents	Sports education institute	R; MR	population	18,5–24,5	F/M	5958	NP
Adegoke et al. [85]	2015	Nigeria	Children/adolescents	School	C; Q	cluster random	10–19	F/M	571	83.97
Vincent-Onabajo et al. [86]	2016	Nigeria	University students	University	C; Q	purposive	20–47	F/M	207	71
Akodu et al. [89]	2016	Nigeria	Filling stations workers	Industry	C; Q	unclear	20–64	F/M	241	95
Odebiyi et al. [90]	2016	Nigeria	Call centre workers	Industry	C; Q	Random	20–49	F/M	120	93.5
Belay et al. [91]	2016	Ethiopia	Nurses	Profession	C; Q	Random	20–60	F	179	91.9

Key: *M* male, *F* female, *SD* standard deviation, *C* cross-sectional, *P* prospective, *R* retrospective, *RR* response rate, *NP* not provided, *I* interview, *E* examination, *Q* questionnaire, *MR* medical records

### Methodological quality of included studies

Twenty-two (33.8%) of the included studies scored 60% or less on the specified critical appraisal tool and were therefore excluded from further analysis [17, 19, 21, 23, 26, 36, 38, 42, 44–46, 49–51, 55, 56, 58, 64, 83, 84, 86, 90]. Sixty-five percent (*n* = 42) of the included studies reported on the validity and/or reliability of their data collection tools (questionnaire, interview or examination) [31, 32, 34, 35, 39, 41–48, 50, 52–70, 81, 83, 85–91]. Only 24 of the included studies (36.9%) provided a case definition for LBP [18, 24, 25, 30–32, 34, 35, 37, 40, 41, 48, 52, 57, 65–69, 81, 83–85, 91]. Table 3 illustrates the methodological appraisal of the included studies.

**Table 3:** Methodological appraisal of included studies (*n* = 65)

Criterion study ID	1	2	3	4	5	6	7	8	9	10	%	MA
Mulimba [17]	+	–	–	+	+	+	–	–	–	–	40	No
Bezzaoucha [18]	+	–	+	+	+	+	–	+	–	+	70	Yes
Harris [19]	–	–	+	+	+	+	–	–	+	+	60	No
Schierhout et al. [20]	+	+	+	+	+	+	–	–	–	+	70	Yes
Mijiyawa et al. [21]	–	–	–	+	–	+	–	–	–	+	30	No
Omokhodion et al. [22]	+	–	+	+	+	+	–	–	+	+	70	Yes
Worku [23]	+	–	+	+	–	+	–	–	+	+	60	No
Wallner-Schlotfeldt et al. [24]	–	+	+	+	+	+	–	+	+	+	80	Yes
Omokhodion et al. [25]	+	+	+	+	+	+	–	+	+	+	90	Yes
Mbaye et al. [26]	–	–	+	+	+	+	–	–	+	+	60	No
Omokhodion et al. [27]	+	+	+	+	+	+	–	–	–	+	70	Yes
Igumbor et al. [28]	+	+	+	+	+	+	–	–	+	+	80	Yes
Omokhodion et al. [29]	+	+	+	+	+	+	–	–	+	+	80	Yes
Govender [30]	+	+	+	+	+	+	–	+	+	+	90	Yes
Prista et al. [31]	+	–	+	+	+	+	+	+	+	+	90	Yes
Fabunmi et al. [32]	+	–	–	+	+	+	+	+	+	+	80	Yes
Sanya et al. [33]	+	–	+	+	+	+	–	–	+	+	70	Yes
Bejia et al. [34]	+	+	+	+	+	+	+	+	+	+	100	Yes
Jordaan et al. [35]	+	+	+	+	+	+	+	+	+	+	100	Yes
Adedoyin et al. [36]	–	+	+	–	+	+	–	–	+	+	60	No
Bejia et al. [37]	+	+	+	+	+	+	–	+	+	+	90	Yes
Van Vuuren et al. [38]	–	–	+	+	+	+	–	–	+	+	60	No
Van Vuuren et al. [39]	+	+	+	+	+	+	+	–	+	+	90	Yes
Galukande et al. [40]	–	+	+	+	+	+	–	+	+	+	80	Yes
Ayanniyi et al. [41]	+	+	+	–	+	+	+	+	+	–	80	Yes
Hill et al. [42]	+	+	–	–	+	+	+	–	–	–	50	No
Bio et al. [43]	+	+	–	+	+	+	+	–	+	+	80	Yes
Balogun and Owoaje [44]	+	–	–	–	+	+	+	–	–	–	40	No
Naidoo and Coopoo [45]	–	+	+	+	+	+	+	–	–	–	60	No
Odebiyi et al. [87]	–	–	+	+	+	+	+	–	+	+	70	Yes
Adegoke et al. [46]	+	–	+	–	+	+	+	–	–	+	50	No
Akinbo et al. [47]	+	+	–	–	+	+	+	–	+	+	70	Yes
Sikiru and Shmaila [48]	+	–	+	+	+	+	+	+	+	+	90	Yes
Booysens et al. [49]	+	–	+	–	+	+	–	–	+	–	50	No
Isa et al. [82]	+	–	–	+	–	+	+	+	+	+	70	Yes
Tinubu et al. [50]	–	–	+	–	+	+	+	–	–	+	50	No
Ouédraogo et al. [51]	–	–	–	+	+	+	–	–	–	+	40	No
Sikiru and Hanifa [52]	–	–	–	+	+	+	+	+	+	+	70	Yes
Abiodun-Solanke et al. [83]	+	+	–	–	+	+	–	–	+	+	60	Yes
Ayanniyi et al. [53]	+	–	+	–	+	+	+	–	+	+	70	Yes
Saidu et al. [64]	–	–	+	–	+	+	+	–	+	–	50	No
Himalowa and Frantz [54]	+	+	+	+	+	+	+	–	–	+	80	Yes
Desai et al. [55]	–	–	–	–	+	+	+	–	+	+	50	No
Noorbhai et al. [56]	+	–	–	–	+	+	+	–	+	+	60	No
Birabi et al. [57]	+	–	–	+	+	+	+	+	+	+	80	Yes
Ogunbode et al. [58]	+	–	–	+	+	+	+	–	–	+	60	No
Oyeyemi et al. [84]	–	–	–	–	+	+	+	+	+	+	60	Yes
Akinpelu et al. [59]	–	–	+	+	+	+	+	–	+	+	70	Yes
Jimoh et al. [60]	+	+	–	+	+	+	+	–	+	+	80	Yes
Madiba et al. [61]	+	–	+	–	+	+	+	–	+	+	70	Yes
Tella et al. [65]	–	–	–	+	+	+	+	+	+	+	70	Yes
Rufa’i et al. [66]	–	+	+	+	+	+	+	+	–	+	80	Yes
Botha et al. [62]	+	–	+	–	+	+	+	–	+	+	70	Yes
El-Soud et al. [63]	+	+	+	+	+	+	+	–	+	–	80	Yes
Chiwaridzo et al. [67]	+	+	+	+	+	+	+	+	+	+	100	Yes
Erick and Smith [68]	+	+	+	+	+	+	+	+	+	+	100	Yes
Mwaka et al. [69]	+	–	+	–	+	+	+	+	+	–	70	Yes
Major-Helstoot et al. [70]	+	+	+	+	+	+	+	–	–	+	80	Yes
Akodu et al. [88]	–	–	+	+	+	+	+	–	+	+	70	Yes
Triki et al. [84]	+	–	–	+	+	+	–	+	–	–	50	No
Adegoke et al. [85]	+	–	+	+	+	+	+	+	+	+	90	Yes
Vincent-Onabajo et al. [86]	–	–	+	+	+	+	+	–	–	+	60	Yes
Akodu et al. [89]	–	–	+	+	+	+	+	–	+	+	70	Yes
Odebiyi et al. [90]	+	–	+	–	+	+	+	–	–	+	60	Yes
Belay et al. [91]	+	–	+	+	+	+	+	+	+	+	90	Yes

Key: + criteria fulfilled; − criteria not fulfilled; *MA* Methodologically acceptable

### Lifetime, annual and point prevalence of LBP among African nations

Lifetime, annual and point prevalence data of LBP among African nations were calculated to provide a summary estimate. Lifetime prevalence pertains to the experience of LBP at any point in the individual’s lifetime; annual prevalence pertains to the experience of LBP at any point in the past 6–12 months; and point prevalence pertains to the experience of LBP at the time of the study’s data collection. For these purposes, only African studies reporting a recall period of lifetime, annual or point prevalence for LBP, were included for analyses.

- *Lifetime prevalence of LBP in Africa*

**Fig. 2:**
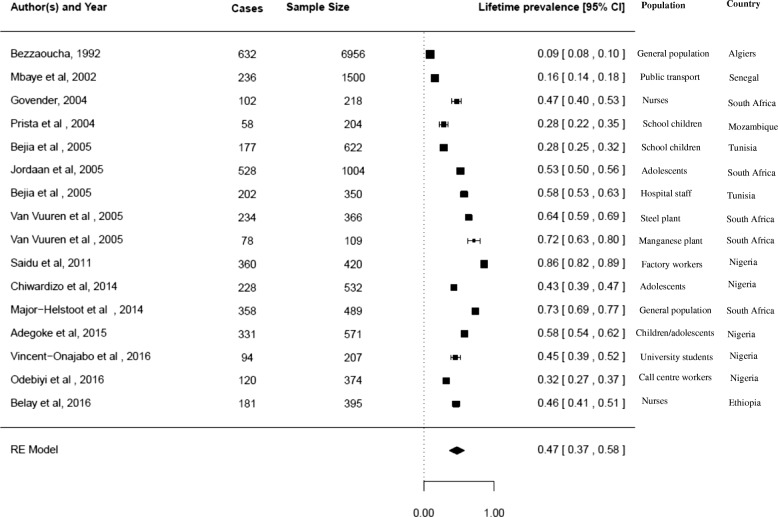
Summary analysis for lifetime prevalence of LBP among African populations

Sixteen studies reported on the lifetime prevalence of LBP in Africa [18, 26, 30, 31, 34, 35, 37, 39, 64, 67, 70, 85, 86, 91]. The lifetime prevalence for LBP in Africa was estimated at 47% (95% CI 37;58). The summary analyses for lifetime prevalence of LBP among Africans is depicted in Fig. 2.

**Fig. 3:**
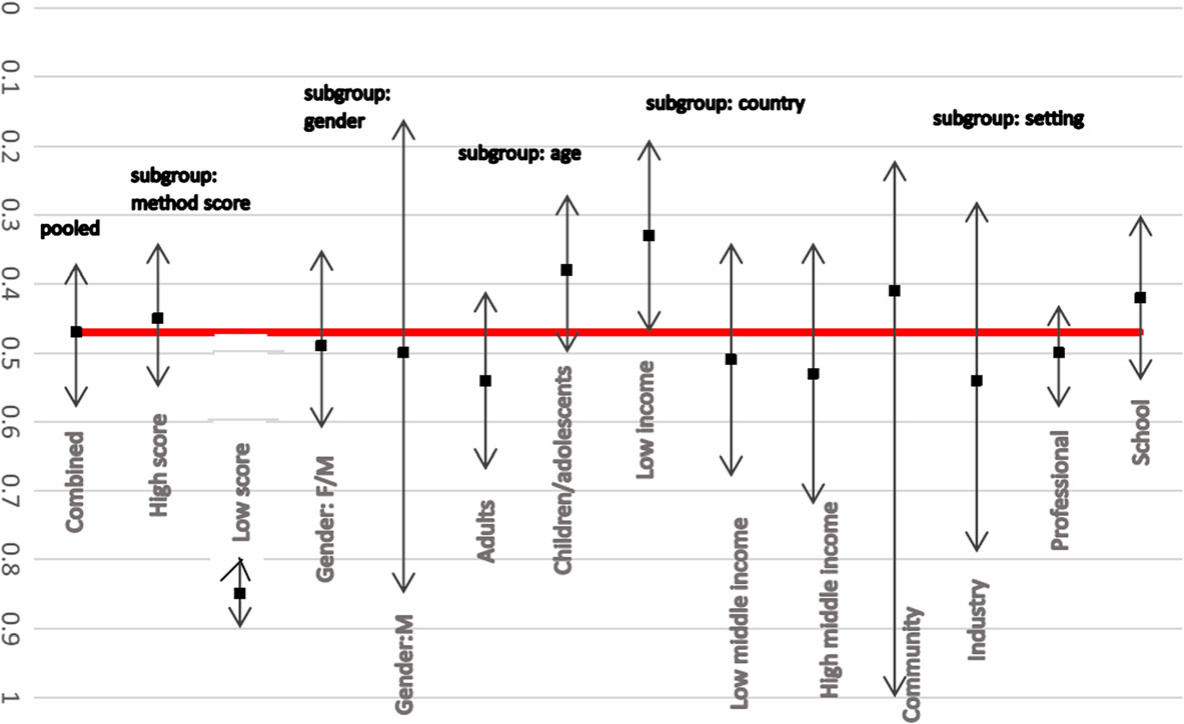
Subgroup analysis for lifetime prevalence of LBP among African nations (*please note: no subgroup data for Gender:F and Setting:Hospital)

Sensitivity and subgroup analyses were conducted to ensure that the exclusion of the poorer methodological quality studies would not have influenced the results significantly if included. Figure 3 illustrates the sensitivity and subgroup analyses conducted for lifetime LBP prevalence among Africans. A significant difference between the summary estimates calculated with only the higher quality studies or only the lower quality studies, compared to all studies (combined) was found.

- *Annual prevalence of LBP in Africa*

**Fig. 4:**
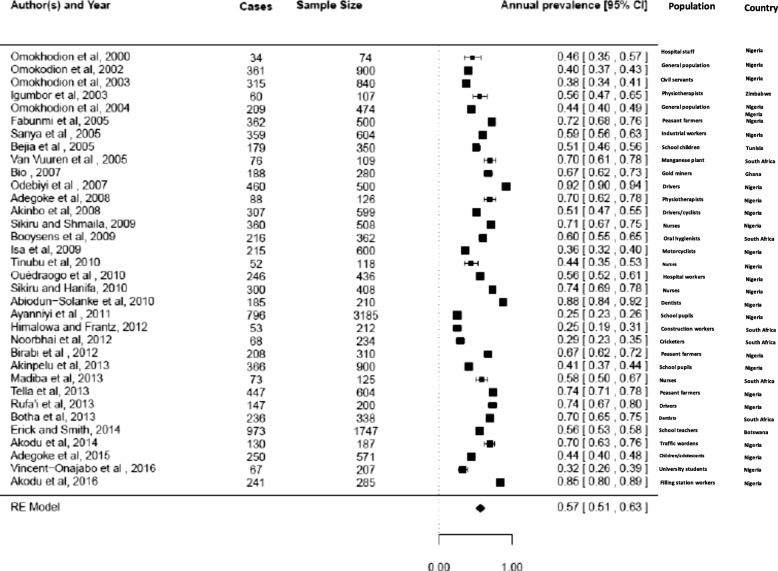
Summary analysis of annual prevalence of LBP among African nations

Thirty-four studies reported on the annual prevalence of LBP in Africa [22, 25, 27–29, 32–34, 37, 39, 43, 46–54, 56, 57, 59, 61, 62, 65, 66, 68, 81, 82, 85–89]. The annual prevalence of LBP in Africa was estimated at 57% (95% CI 51;63). The summary analyses for annual prevalence of LBP among Africans is depicted in Fig. 4.

**Fig. 5:**
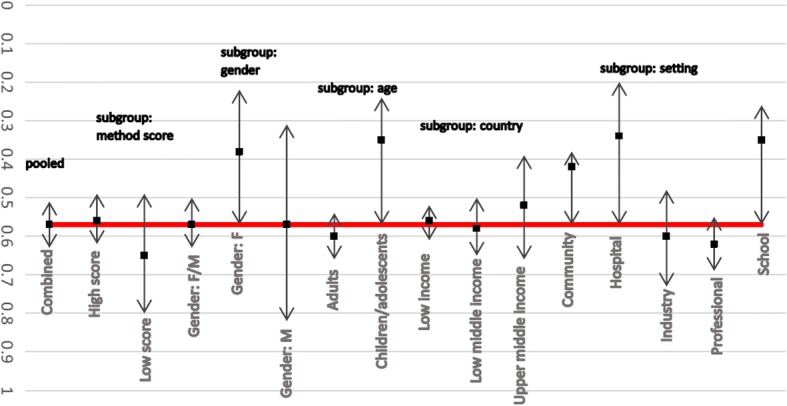
Subgroup analysis of annual prevalence of LBP among African nations

Figure 5 illustrates the sensitivity and subgroup analyses for annual LBP prevalence among African nations. No significant differences between the summary estimates calculated with only the higher quality studies or only the lower quality studies, compared to all studies (combined) were found.

- *Point prevalence of LBP in Africa*

**Fig. 6:**
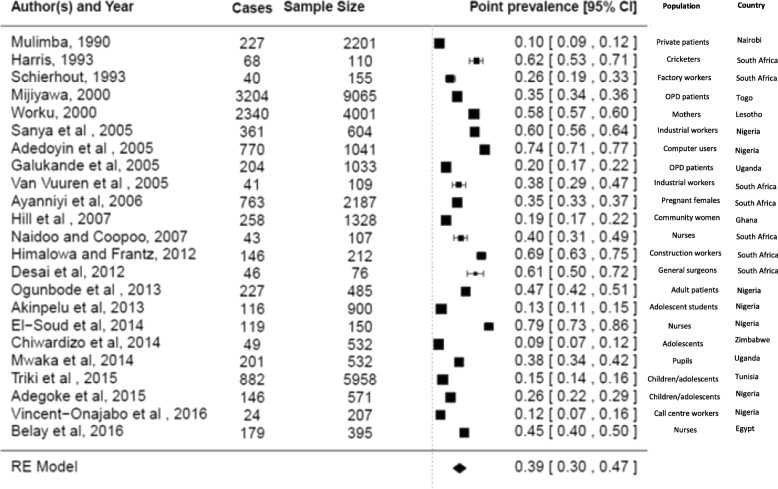
Summary analysis of point prevalence among African nations

Twenty-three studies reported on point prevalence of LBP in Africa [17, 19–21, 23, 33, 39–42, 45, 54, 55, 58, 59, 63, 67, 69, 84–86, 91]. The point prevalence of LBP in Africa was estimated at 39% (95% CI 30;47). The summary analyses for point prevalence of LBP among Africans is depicted in Fig. 6.

**Fig. 7:**
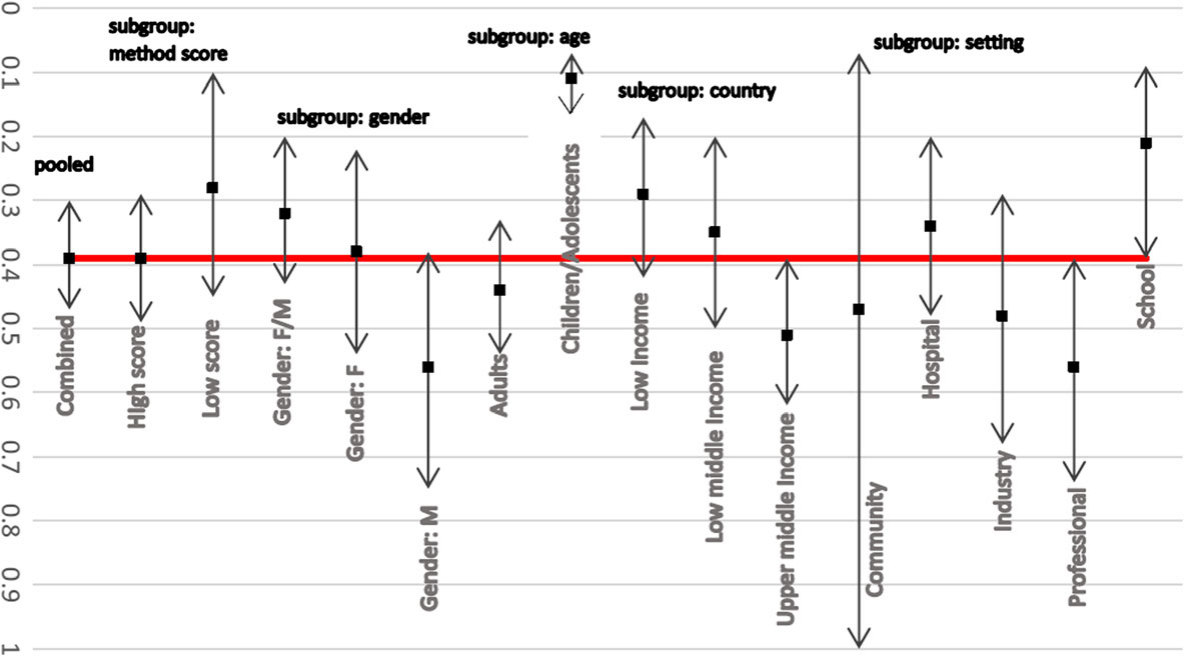
Subgroup analysis of point prevalence of LBP among African nations

Figure 7 illustrates the subgroup and sensitivity analyses for point LBP prevalence among Africans. No significant differences between the summary estimates calculated with only the higher quality studies or only the lower quality studies, compared to all studies (combined) were found.

### Publication bias

Duval and Tweedie’s “Trim and Fill” method was used to assess publication bias [80]. Under the random effects model the point estimate and 95% confidence interval for the combined studies is 0.49 (95% CI 0.39, 0.57). Using Trim and Fill the imputed point estimate is 0.31 (95% CI 0.24, 0.39). The method suggests that a total of 13 studies may be missing from this review.

## Discussion

This paper provides an updated synthesis of the literature into the prevalence of LBP among African populations. The current review indicates that although a number of years have passed after our initial review [10], LBP remains a health concern in Africa.

Meta-analyses of the observational data collected from the eligible studies provides a summary estimate of the lifetime, annual and point prevalence. Lifetime, annual and point prevalence of LBP among African populations was found to be higher than recently reported estimates for global LBP prevalence [2, 4, 5]. The global prevalence of LBP reported by Hoy et al. in 2012 was calculated from a total of 165 studies conducted in 54 countries around the globe (developed and developing countries), over a period of 29 years [2]. In our review, the point prevalence of LBP among Africans was estimated at 39% (95% CI 30;47), which is considerably higher than the global LBP prevalence estimate (18.3%) reported by Hoy et al. [2]. Similarly, the annual prevalence for LBP among Africans (57%; 95% CI 51;63) found in our review was substantially higher than the global annual LBP prevalence (38.5%) reported by Hoy et al. [2]. The lifetime prevalence for LBP among Africans (47%; 95% CI 37;58) was also found to be considerably higher than the estimates (38.9%) reported by Hoy et al. [2]. The summary estimates found in this review were compared specifically to North American and Western European countries. It was found that the point LBP prevalence among Africans was substantially higher than estimates provided for Canada (28.7%), Denmark (12–13.7%) and Sweden (23.2%), and was comparable to Germany (39.2) and Belgium (33%) [5]. One year LBP prevalence among Africans was considerably higher than Spain (20%), and on par with Denmark (56%) and Ukraine (50.3%) [5]. The findings of this review therefore reiterates the fact that LBP is a burden and is therefore a public health concern among developing nations in Africa [4, 7, 8]. Despite the high burden, LBP remains a lower priority compared to epidemics such as HIV/AIDS in Africa [3]. African healthcare budgets and systems may be generally ill-prepared to deal with the management of LBP which could partly explain the high LBP prevalence among African populations [4, 9, 10, 71]. The successful development and implementation of strategies and policies to address the burden of LBP in poorer countries or countries with emerging economies, like those in Africa, is therefore warranted [9].

The lifetime, annual and point prevalence of LBP was estimated to be higher among African adults compared to African children and adolescents. This finding confirms that similarly to developed nations, the prevalence of LBP among Africans increases with age [1, 2, 6]. These summary estimates for annual and lifetime LBP prevalence among African children and adolescents were however found to be higher than estimates reported for the United Kingdom (15.6–24%), Finland (9.7%), and Iran (15%), and comparable to Iceland (34%) and Denmark (32.4%) [5], although point prevalence was found to be lower or on par (11%). Of concern is that the early onset of LBP in childhood or adolescents is a risk factor for developing chronic LBP later in life [53, 72], and once the younger generation become the working class, the ongoing pain and related disability will ultimately affect work productivity and the economy of a country [1, 3, 53]. Therefore, in developing countries or countries with emerging economies like African countries, where budgets are already stringent [10], it would make sense to implement effective prevention strategies to the risk of developing LBP in childhood and/or adolescence, in anticipation of the future economically drain LBP may place on the individual, the industry and the state [53]. Future studies should therefore investigate the factors which lead to the early onset of LBP among African children and adolescents and develop prevention strategies which are effective, feasible and accessible to all people living in rural and urban areas of Africa.

The findings of this review also clearly show a notable difference in point and annual LBP prevalence of close to 20% between African males and females, with males reporting a higher prevalence. These results indicate a reverse gender pattern compared to global trends which generally indicate that females experience a higher prevalence [92]. What is interesting about this finding is that within most African cultures, African males actually tend to under-report health issues as it is perceived to reduce their masculinity [93]. A higher prevalence for African females would therefore have been expected. However, this said, these findings may also be linked to the fact that half of the studies on industry included mostly males or males only, whereas the workers included in the professional subgroup included more females. Since industry-related jobs include more intense physical labour, an over-representation of males may have therefore resulted.

### Epidemiologic and methodological challenges in conducting LBP prevalence reviews among African populations

The review process highlighted a number of challenges related to conducting, sourcing and pooling relevant epidemiologic data in Africa. One of the first methodological challenges when conducting such a review, was the uncertainty of whether all relevant data were included in the review. This is because a number of African research studies may not have been published in journals which are indexed in accessible and commonly-used international databases [73, 74]. Many African LBP studies are published in local journals or as a postgraduate thesis, and not all African universities may have information technology systems which allow online access to their postgraduate theses [74]. Data may therefore only be available in the local university libraries. Furthermore, African LBP researchers may not have the opportunity to publish in open access journals due to the associated high publication costs [73–75], which leads to difficulty in publishing, as well as accessing and retrieving such publications. The inclusion of all relevant African literature on LBP prevalence can therefore not be guaranteed.

Another challenge in conducting this review is the fact that Africa is riddled by huge economic inequality between countries. We found that most studies were conducted in Nigeria and South Africa, which have the strongest economies in Africa and are currently ranked first and third, respectively in terms of Gross Domestic Product [75]. In these relatively more affluent countries, factors such as economic growth and urbanisation have already followed patterns noted in the developed world and this could have an effect on LBP occurrence and reporting [75]. While research fields such as HIV/AIDS and TB in Africa are well funded by international bodies, this is not the case with LBP research [3]. LBP research in poorer African countries is consequently not possible or encouraged due to prioritisation of research funding towards other pressing health issues. The economic inequality between African countries could therefore have biased our review findings to more affluent countries.

The poor methodological quality of included studies posed another challenge in conducting this review since just over 60% of the studies could be used in the analyses. Of concern was that most of the shortcomings in the methods reported by the poor quality studies could have been avoided. Similarly, to the previous review [10], and other reviews [2], the poor quality studies in this review generally did not provide a definition of LBP, lacked adequate representation of the population, did not provide response rates or drop-out rates, and neglected to use reliable and/valid instruments (be it a questionnaire, interview, or examination) for collection of data. According to Dionne et al., it is highly recommended that epidemiologic studies should at least provide the case definition used in establishing the prevalence of LBP in a specified population [76]. In addition, this case definition for LBP should be standardized to ensure that greater comparisons between countries (developed or developing) can be made [76, 77], for a greater understanding of LBP to be gained [2]. The validity and reliability of instruments should also be established prior to their administration in a specific population to ensure accurate estimates of prevalence [78]. One important area to address is the development of a valid and reliable LBP measurement instrument which should ideally take context and culture into account. Furthermore, improved collaboration between researchers in different African countries, will facilitate standardization of measuring LBP among Africans to assist with comparisons across countries as well as meta-analytical approaches. It is therefore recommended that future studies prioritize conducting studies with improved methodological quality, provide and use a standardized case definition of LBP, and report essential information, which will lead to accurate assessment, interpretation, translation and comparison of results across studies [79].

Lastly, although measures were taken to ensure that the heterogeneity among studies was considered during meta-analyses, the summary estimates provided in this review should still be viewed with caution [2]. Heterogeneity in observational studies is however expected [76, 77, 79], since populations, and even cultural groups within a specific population, inherently differ [94]. More specifically, heterogeneity of LBP data remains considerable across studies due to the lack of a standardized or universal case definition for LBP [76, 77]. For this reason, the pooling and comparison of LBP data based on different definitions is a challenge on its own, regardless of population and other study characteristic variability [77].

## Conclusion

Since the original review was published in 2007, a number of epidemiologic studies into the prevalence of LBP in Africa have emerged. This review found that the lifetime, annual and point prevalence of LBP among African nations, was higher than the global LBP prevalence reported. Prevention strategies addressing the early onset of LBP among the youth would most likely be the answer to addressing the burden of LBP on future economies in Africa. Caution must however be taken when interpreting the summary estimates provided in this current review, since high heterogeneity, which is expected, was displayed among the included studies. Furthermore, due to the poor methodological quality found among many of the included studies, the over-representation of more affluent African countries and the difficulty in sourcing and retrieving potential African studies, it is recommended that future African LBP researchers conduct methodologically robust studies and report their findings in accessible resources.

## Abbreviations

CI: Confidence interval
GBD: Global burden of disease
LBP: Low back pain
LMICs: Lower and middle income countries
WHO: World Health Organization
YLDs: Years lived with disability
